# Co-incorporation of manure and inorganic fertilizer improves leaf physiological traits, rice production and soil functionality in a paddy field

**DOI:** 10.1038/s41598-021-89246-9

**Published:** 2021-05-11

**Authors:** Anas Iqbal, Liang He, Izhar Ali, Saif Ullah, Aziz Khan, Kashif Akhtar, Shangqin Wei, Shah Fahad, Rayyan Khan, Ligeng Jiang

**Affiliations:** 1 College of Life Science and Technology, Guangxi University, Nanning, 530004 China; 2 Key Laboratory of Crop Cultivation and Farming Systems College of Agriculture, Guangxi University, Nanning, 530004 China; 3 Institute of Nuclear Agricultural Sciences, College of Agriculture and Biotechnology, Zhejiang University, Hangzhou, 310058 China; 4 Department of Agronomy, The University of Haripur, Khyber Pakhtunkhwa, Pakistan; 5 Tobacco Research Institute, Chinese Academy of Agricultural Science, Qingdao, 266101 China

**Keywords:** Plant sciences, Environmental sciences

## Abstract

The combined use of organic manure and chemical fertilizer (CF) is considered to be a good method for sustaining high crop yields and improving soil quality. We performed a field experiment in 2019 at the research station of Guanxi University, to investigate the effects of cattle manure (CM) and poultry manure (PM) combined with CF on soil physical and biochemical properties, rice dry matter (DM) and nitrogen (N) accumulation and grain yield. We also evaluated differences in pre-and post-anthesis DM and N accumulation and their contributions to grain yield. The experiment consisted of six treatments: no N fertilizer (T_1_), 100% CF (T_2_), 60% CM + 40% CF (T_3_), 30% CM + 70% CF (T_4_), 60% PM + 40% CF (T_5_), and 30% PM + 70% CF (T_6_). All CF and organic manure treatments provided a total N of 150 kg ha^−1^. Results showed that the treatment T_6_ increased leaf net photosynthetic rate (*Pn*) by 11% and 13%, chlorophyll content by 13% and 15%, total biomass by 9% and 11% and grain yield by 11% and 17% in the early and late season, respectively, compared with T_2_. Similarly, the integrated manure and CF treatments improved post-antheis DM accumulation and soil properties, such as bulk density, organic carbon, total N, microbial biomass carbon (MBC) and microbial biomass nitrogen (MBN) relative to the CF-only treatments. Interestingly, increases in post-anthesis DM and N accumulation were further supported by enhanced leaf *Pn* and activity of N-metabolizing enzyme during the grain-filling period. Improvement in *Pn* and N-metabolizing enzyme activity were due to mainly improved soil quality in the combined manure and synthetic fertilizer treatments. Redundancy analysis (RDA) showed a strong relationship between grain yield and soil properties, and a stronger relationship was noted with soil MBC and MBN. Conclusively, a combination of 30% N from PM or CM with 70% N from CF is a promising option for improving soil quality and rice yield.

## Introduction

Rice (*Oryza sativa* L.) is the main staple food eaten by half of the world’s inhabitants and almost 60% of the Chinese population^1,2^. China is the leading globle producer and consumer of rice^1^. Nitrogen (N) is essential for plant growth, and its application influences crop yield by establishing and maintaining photosynthetic and sink capacities^3,4^. The current farming system is heavily reliant on synthetic N fertilizers to achieve higher yields. However, crop yield does not necessarily increase linearly with N fertilizer input^5,6^. Excessive N application causes significant environmental problems, such as increased greenhouse gas emission, groundwater contamination, and surface water eutrophication^7,8^. Long-term use of chemical N fertilizer has also caused the acidification, degradation, and compaction of arable soils, thereby suppressing plant growth and productivity^9,10^. Moreover, the extreme use of N and phosphorus fertilizers also reduces the soil microbial population and increases soil acidity^11,12^. Therefore, the continued reliance on synthetic N fertilizer for cereal crop production is not sustainable. Proper management practices are needed to improve N use efficiency, and reduce the harmful effects of CFs on soil health, and ensure sustainable rice production.

Organic manure derived from animal waste holds great promise, for maintaining soil health and fertility, protecting soil biodiversity, and improving crop production; organic fertilizers have a clear advantage over CFs in many aspects^13–15^. Organic fertilizer has greater organic matter and richer nutrient contents; it improves soil physicochemical and biological properties primarily by enhancing soil structure and reducing soil bulk density^16–18^. The slow and steady N release from organic fertilizers is a benefit to sole CF application in achieving higher N use efficiency , crop yield, and grain quality^19,20,21^. However, organic manure is relatively low in nutrient content, and its release rate is generally too slow to meet plant requirements in a short time period. Thus, the combined application of organic manure and CF has proven to be a promising option in improving and maintaining soil fertility and crop production than the use of either amendments or alone^20,22^. Although the advantages of organic manure fertilization are generally recognized, its optimal application ratio with CF remains unclear.

Plant leaves play two major roles, particularly during the grain filling period of cereal crops: they are the primary photosynthetic organs necessary for the production of DM, and they are the main source of grain filling^23^. Delayed leaf senescence promotes relatively high photosynthetic rates and is desirable for producing the highest cereal yield that can be synchronized with sufficient soil N availability during the grain-filling period^24,25^. Geng et al.^26^ reported that organic manure application improved leaf photosynthetic capacity and chlorophyll content during the reproductive period because of the slow and steady release of mineral nutrients from manure during the entire growing season, particularly during the grain-filling period. Nitrogen is taken up and assimilated through N-metabolizing enzymatic pathways, that include nitrate reductase (NR), glutamine synthetase (GS), and glutamate oxoglutarate aminotransferase (GOGAT)^27^. Maintenance of N recovery by N metabolizing enzymes not only enhances the plant’s photosynthetic ability but also prolongs the stay-green period leaves^28,29^.

Crops grain yield derives from two sources, the translocation of plant DM accumulated pre-anthesis and post-anthesis DM production^30^. Previous research has demonstrated the value of pre-anthesis plant DM accumulation and translocation, which can contribute to higher grain yields^30^. Furthermore, it was shown that 69% of straw N and 84% of non-structural carbohydrates accrued pre-anthesis could be translocated to the grains, although this depends on the sowing conditions and cultivar^31^. Post-anthesis DM production may be a good contributor to cereal grain yield, according to recent evidence^32,33^. However, due to a lack of information, further study into the relationship between post-anthesis DM production and grain yield is required. In the present study, we hypothesized that the combined use of organic and inorganic fertilizers would improve soil physiochemical and biochemical properties, which would in turn increase plant nutrient uptake and accumulation. We also predicted that the slow and steady release of mineral nutrients from organic fertilizers during the entire plant growth period would improve leaf physiological activity, post-anthesis DM, and N accumulation, thereby increasing grain yield. The objectives of the study were: (1) to determine the effects of organic and inorganic N fertilizer combinations on soil physical and biochemical properties; (2) to assess the effect of integrated fertilization on rice leaf physiology, biomass and grain yield; and (3) to evaluate the difference in pre-and post-anthesis N and DM accumulation and its relationship to rice grain yield.

## Results

### Soil properties

The combined application of organic and inorganic fertilizer and the different seasons had significant effects on soil physical and chemical properties such as bulk density (BD), pH, soil organic carbon (SOC), total nitrogen (TN), available nitrogen (AN), available potassium (AK), and availible phosphorus (AP) up to 20 cm (Table 1). The treatments showed the same behavior across both seasons. In the CF-only treatment (T_2_), soil pH (5.92 and 5.90), SOC (14.86 and 14.66 g kg^−1^), TN (1.45 and 1.43 g kg^−1^), AN (138.5 and 137.5 mg kg^−1^), AP (23.5 and 24.5 mg kg^−1^) and AK (226.5 and 231.2 mg kg^−1^) were recorded in the early and late seasons, respectively. Compared with T_2_, the combined treatment T_3_ increased soil pH by (4.5% and 7.2%), SOC (13% and 19%), TN (14% and 25%), AN (11% and 18%), AP (14% and 22%), and AK (22% and 33%) during the early and late seasons, respectively. Treatment T_3_ also considerably reduced the soil BD relative to T_2_ by 6% in the early season and 9% in the late season. Soil pH, SOC, TN, AN, AP, and AK in T_3_ increased by 60%, 46%, 78%, 63%, 57%, and 50%, respectively, in the late season compared with the early season. Soil properties in the treatment T_5_ did not differ significantly from those in T_3_. Moreover, treatments T_4_ and T_6_ also significantly improved soil physical and chemical properties relative to T_2_ Lower values for soil parameters were noted in the non N-treated plots.

**Table 1:** Changes in soil physical and checmial properties under the combined organic and inorganic N fertilization.

Treatment	BD (g cm^-3^)	pH (water)	SOC (g kg^−1^)	SOM (g kg^−1^)	TN (g kg^−1^)	AN (mg kg-1)	AP (mg kg^−1^)	AK (mg kg^−1^)
**Early season**								
T_1_	1.36 ± 0.01 a	5.97 ± 0.32 c	13.53 ± 0.85 d	25.0 ± 2.05 d	1.40 ± 0.08 c	132.82 ± 6.55 d	23.23 ± 1.54c	225.22 ± 10.55 c
T_2_	1.36 ± 0.02 a	5.95 ± 0.24 c	14.10 ± 1.01 d	25.01 ± 1.52 d	1.41 ± 0.03 c	138.53 ± 8.05 c	23.54 ± 2.00 c	226.54 ± 12.36 c
T_3_	1.24 ± 0.02 c	6.10 ± 0.34 a	16.81 ± 0.44 a	29.20 ± 0.85 a	1.63 ± 0.02 a	154.38 ± 10.11 a	26.59 ± 1.05 a	276.53 ± 11.50 a
T_4_	1.29 ± 0.04 b	6.00 ± 0.50 b	15.41 ± 0.81 b	28.15 ± 1.58 c	1.54 ± 0.07 b	148.87 ± 9.44 b	24.81 ± 2.15 b	266.80 ± 15.50 b
T_5_	1.25 ± 0.01 c	6.11 ± 0.30 a	16.69 ± 1.44 a	28.60 ± 2.87 a	1.65 ± 0.10 a	155.40 ± 5.05 a	26.87 ± 1.22 a	274.28 ± 7.55 a
T_6_	1.30 ± 0.06 b	6.03 ± 0.41 b	15.01 ± 0.28 c	26.42 ± 1.81 c	1.52 ± 0.04 b	149.60 ± 8.57 b	24.59 ± 1.88 b	265.60 ± 9.33 b
Average	1.30 a	6.02 b	14.80 b	27.17 b	1.53 b	146.60 b	24.94 b	255.83 b
** Late season**								
T_1_	1.36 ± 0.04 a	5.96 ± 0.21 c	13.44 ± 1.08 d	24.85 ± 1.74 d	1.39 ± 0.02d	133.14 ± 12.01 d	24.28 ± 3.01 c	226.42 ± 15.57 d
T_2_	1.35 ± 0.03 a	5.90 ± 0.12 d	14.66 ± 0.35 c	25.22 ± 2.35 c	1.43 ± 0.05 c	137.55 ± 5.25 c	24.80 ± 2.43 c	231.21 ± 8.88 c
T_3_	1.17 ± 0.01d	6.28 ± 0.62 a	17.68 ± 0.42 a	29.10 ± 1.85 a	1.83 ± 0.04 a	167.79 ± 6.78 a	30.31 ± 2.55 a	308.24 ± 10.76 a
T_4_	1.21 ± 0.05 b	6.19 ± 0.40 b	16.21 ± 0.85 b	28.74 ± 0.95 b	1.71 ± 0.03 b	156.56 ± 11.05 b	27.78 ± 1.42 b	286.52 ± 6.55 b
T_5_	1.20 ± 0.07 c	6.26 ± 0.84 a	17.56 ± 0.70 a	31.92 ± 3.05 a	1.81 ± 0.02 a	168.80 ± 10.56 a	28.45 ± 2.77 a	303.25 ± 16.20 a
T_6_	1.23 ± 0.02b	6.18 ± 0.33 b	16.31 ± 1.22 b	28.62 ± 2.11 b	1.72 ± 0.04 b	158.25 ± 5.33 b	30.54 ± 3.11 b	284.52 ± 9.30 b
Average	1.25 b	6.12 a	15.98 a	28.08 a	1.65 a	162.68 a	27.69 a	273.36 a
**ANOVA**								
Treatment (T)	**	*	**	**	**	*	*	*
Season (S)	*	*	*	*	*	*	*	*
T × S	ns	ns	ns	ns	ns	ns	ns	ns

Note: *T*_1_, no N fertilizer, *T*_2_ 100% CF, *T*_3_ 60% CM + 40% CF, *T*_4_ 30% CM + 70% CF, *T*_5_ 60% PM + 40% CF, *T*_6_ 30% PM + 70% CF, *BD* bulk density, *SOC* soil organic carbon, *SOM* soil organic matter, *TN* total nitrogen, *AP* available phosphorous, *AK* available potassium. Values followed by the same letters, within column, are not significantly different at *p* ≤ 0.05. Mean values (n = 3) ± SE. ns: non significant, *p < 0.05, **p < 0.05.

### Microbial biomass C and N

**Figure 1:**
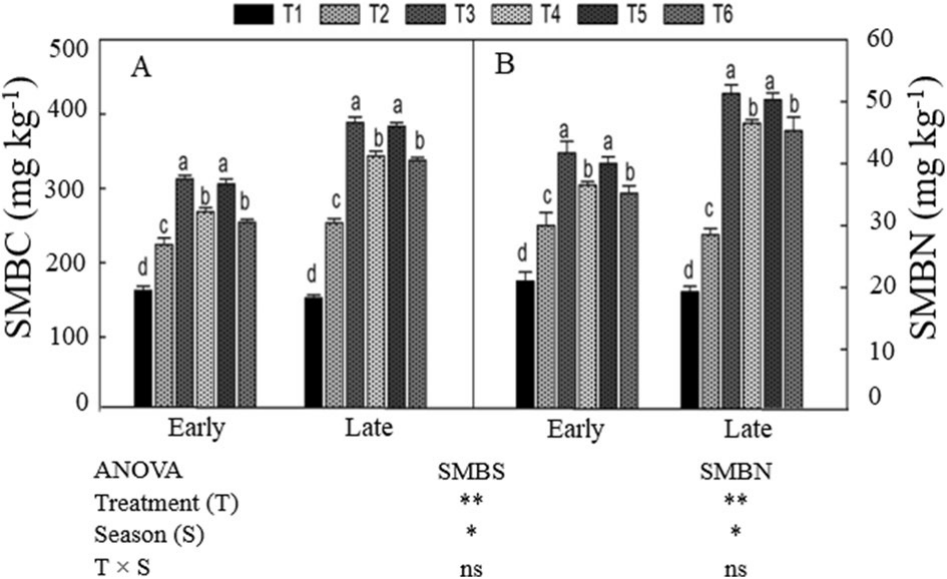
Changes in soil microbial biomass C (SMBC) in the early and late season (**A**) and N (SMBN) during early and late (**B**) under combined organic and inorganic N fertilization. ns: non significant, *p < 0.05, **p < 0.05. For treatment details please see Table 1.

Soil MBC and MBN differed significantly among treatments in both seasons, as shown in Fig. 1. The integrated use of organic manure and CF significantly enhanced soil MBC and MBN in both seasons compared with CF-only fertilization. In the both seasons, the treatments showed the same trend. Compared with CF-only (T_2_), the T_3_ treatment improved soil MBC by (14% and 26%) and MBN (11% and 19%) in the early and late seasons, respectively. Relative to the early season, MBC and MBN in treatment T_3_ increased by 85% and 72%, respectively, in the late seasons. However, MBC and MBN did not differ significantly between T_3_ and T_5_. The treatments T_4_ and T_6_ also significantly improved soil microbial C and N compared with CF-only fertilization and non N-treated plots.

### Net photosynthetic efficiency and chlorophyll content of the flag leaf

**Figure 2:**
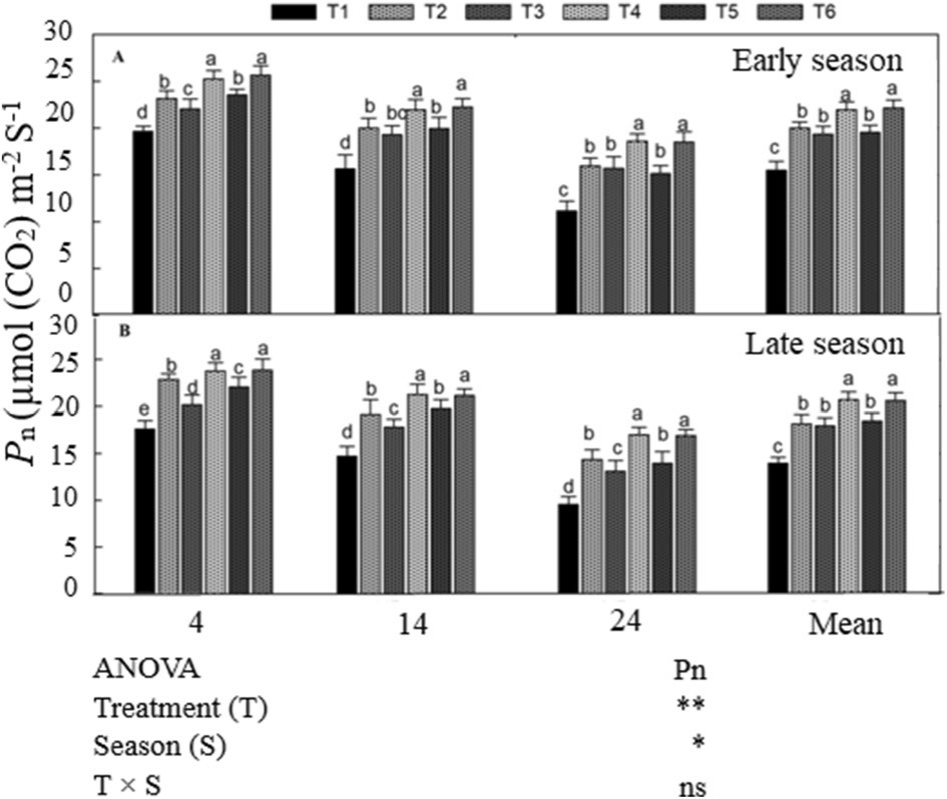
Changes in leaf net photosynthesis rate at (4, 14, and 24  DAA) during grain filling period both early (**A**) and late season (**B**) under the combined organic and inorganic N fertilization. Vertical bars represent the standard error of mean. Different letters above the column indicate significance at the (*P* < 0.05). ns: non significant, *p < 0.05, **p < 0.05. For treatment details please see Table 1.

**Figure 3:**
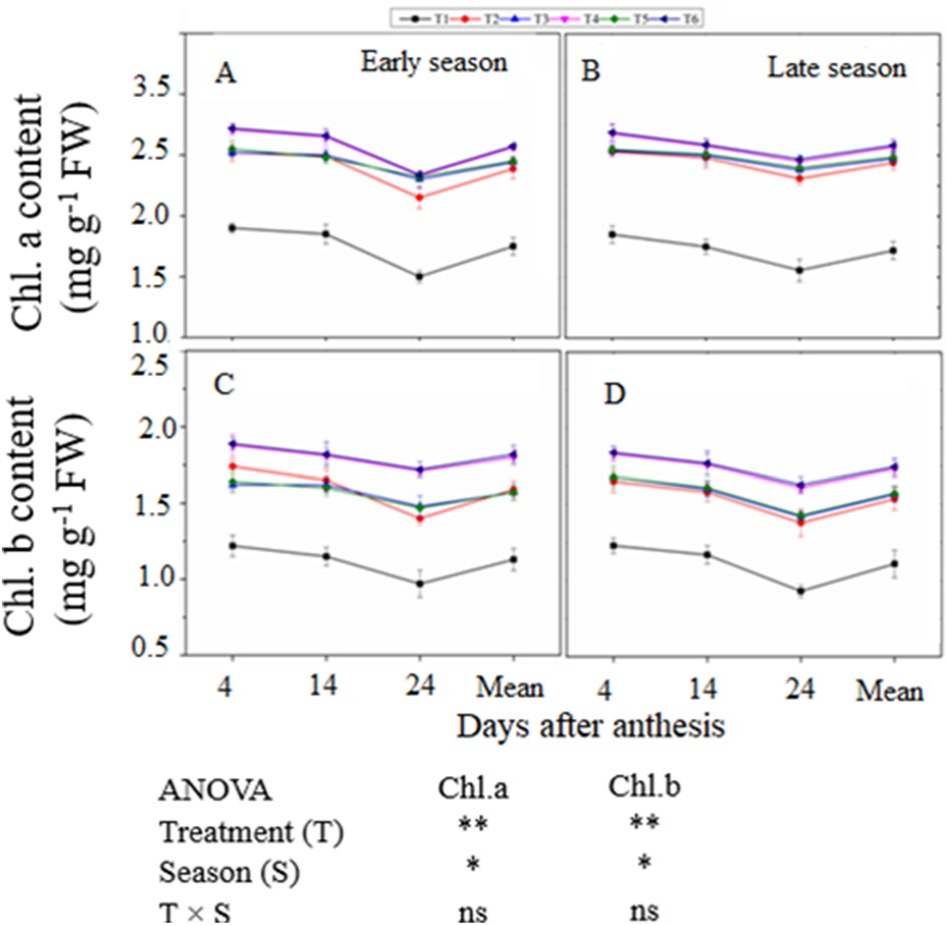
Leaf chlorophyll content a and b at 4, 14, and 24 days after anthesis during grain filling at early (**A**,**C**) and late (**B**,**D**) season in response to combined organic and inorganic N fertilization. Vertical bars represent the standard error of means. Different letters above the column indicate significance at (*P* < 0.05). Note: Chl.a–Chlorophyll a, Chl.b–Chlorophyll b, FW–Fresh leaf weight. ns: non significant, *p < 0.05, **p < 0.05. For treatment details please see Table 1.

The combination of manure and chemical N fertilizer significantly improved *Pn,* chlorophyll a (Chl a), and chlorophyll b (Chl b) content during the grain filling period in both seasons compared with the CF-only application (Figs. 2, 3). Leaf *Pn*, Chl a, and Chl b declined with increasing days after anthesis (DAA). The treatments exhibited the same trend in both seasons. Averaged across DAA, T_6_ treatment increased *Pn* by 11% and 13%, Chl a by 12% and 14%, and Chl b by 14% and 16% in the early and late seasons, respectively, compared with T_2_. However, T_4_ was noted statistically (*P* < *0.05*) non-significant with T_6_. Similarly, the combined treatments T_3_ and T_5_ enhanced leaf *Pn* and Chl content compared with non N-treated plots, but did not differ significantly from T_2_ in either season.

### Activity of N-metabolizing enzymes

**Figure 4:**
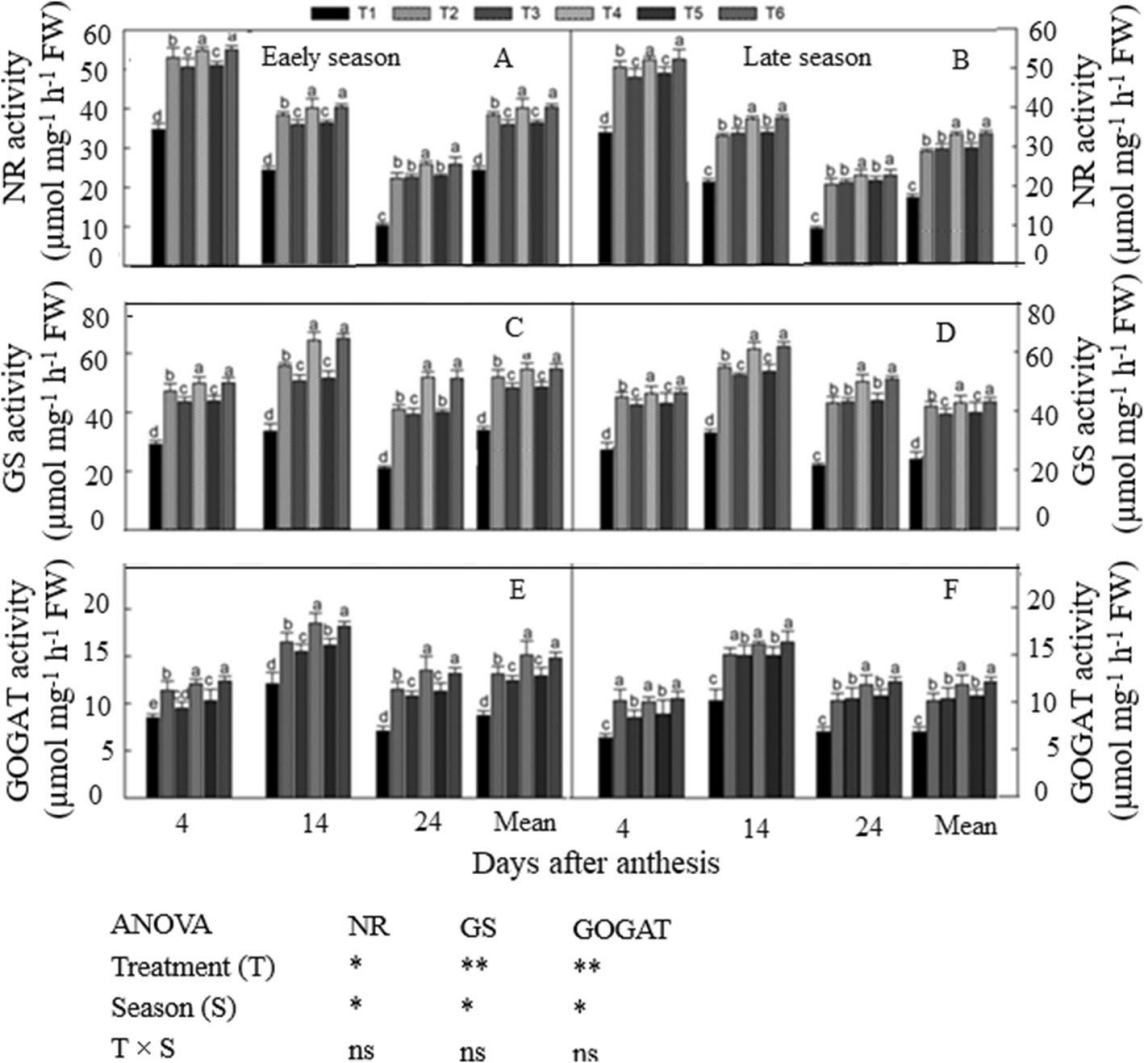
Changes in N metabolism enzyme activities at 4,1 4, and 24 days after anthesis during grain filling period, NR,GS and GOGAT at early season (**A**–**C**–**E**) and late season (**B**–**D**–**F**) in response to combined organic and inorganic N fertilizer application. Vertical bar represents the standard error of mean. Different letters above the column indicate statistical significance at the (*P* < 0.05). Note: NR–nitrate reductase, GS–glutamine synthetase, GOGAT–glutamine oxoglutarate aminotransferase. ns: non significant, *p < 0.05, **p < 0.05. For treatment details please see Table 1.

The activity of N-metabolizing enzymes such as NR, GS, and GOGAT during the grain filling period were significantly affected by the combined application of manure and chemical fertilizer and different seasons (Fig. 4). Higher activity of N-metabolizing enzymes was noted in N-treated plants, wheareas lower activity was observed in non N-treated plants during both seasons (Fig. 4A–F). N-metabolizing enzyme activity showed the same behavior in both seasons. NR activity decreased durig the the grain filling period; it reached a maximum at 4 DAA, then slowly decreased and was lowest at 24 DAA (Fig. 4A, B). Averaged across DAA, the NR activity of T_6_ was 10% and 14% higher than that of T_2_ in the early and late seasons, respectively. NR activity did not differ significantly among T_4_ and T_6_. Similarly, T_3_ and T_5_ also improved NR activity, and the lowest values were observed in nonN-treated plots.

By contrast, GS and GOGAT activities first increased and then decreased during the grain filling period; highest at 14 DAA and lowest at 24 DAA (Fig. 4C–F). Across the grain filling period, GS activity was (13% and 17%) and GOGAT was (11% and 16%) higher in T_6_ than in T_2_ in the early and late seasons, respectively. However, no significant differences were observed among T_4_ and T_6_. The combined fertilization treatments T_3_ and T_5_ had significantly higher values of GS and GOGAT activity than the non N-treated plots.

### Accumulation and translocation of DM and N

Dry matter and N accumulation reflect plant growth and metabolic capacity and ultimately control the economic yield. In the present study, the accumulation of DM and N increased progressively with plant growth and attained maximum values at plant maturity. DM and N accumulation differed significantly among fertilizer treatments and seasons, as shown in Table 2. DM accumulation was 9% and 11% higher in T_6_ than in T_2_ at maturity in the early and late seasons, respectively, and N accumulation was 10% and 12% higher in T_6_. DM and N accumulation in T_6_ were 22% and 20% higher in the late season than in the early season. DM and N did not differ significantly between T_4_ and T_6_. The combined treatments T_3_ and T_5_ also improved DM and N accumulation but did not differ significantly from T_2_. The lowest values of DM and N accumulation were observed in non-N treated plots in both seasons.

**Table 2:** Pre-and post-anthesis dry matter and nitrogen accumulation and translocation under the combined organic manure and inorganic fertilization.

Treatment	DMA (g m^−2^)			DMT (g m^−2^)	NA (g m^−2^)			NT (g m^−2^)
	Ant	Mat	Post-a DMA		Ant	Mat	Post-a NA	
**Early season**								
T_1_	648 ± 14.52 d	956 ± 16.02 d	308 ± 14.20 c	214 ± 8.82 c	6.27 ± 0.85 c	8.84 ± 0.82 c	2.64 ± 0.32 c	4.42 ± 0.44 b
T_2_	998 ± 17.21 b	1480 ± 20.52 b	482 ± 11.08 b	249 ± 11.52 a	10.12 ± 1.12 b	14.30 ± 1.44b	4.18 ± 0.82b	6.28 ± 0.30 a
T_3_	969 ± 16.54 b	1399 ± 18.22 c	430 ± 14.54 b	241 ± 13.84ab	10.45 ± 0.92 a	14.78 ± 0.82 b	4.33 ± 0.22 b	6.24 ± 0.34 a
T_4_	1092 ± 11.52 a	1598 ± 16.50 a	506 ± 12.58 a	266 ± 13.00 a	11.40 ± 0.58 a	16.11 ± 1.12 a	4.71 ± 0.70 a	6.63 ± 0.80 a
T_5_	909 ± 18.00 c	1370 ± 19.82 c	461 ± 18.52 b	236 ± 15.10 b	10.75 ± 0.82 b	14.71 ± 0.62 b	3.96 ± 0.14 b	6.78 ± 0.34 a
T_6_	1094 ± 16.12 a	1618 ± 21.30 a	524 ± 10.12 a	251 ± 9.85 ab	11.50 ± 1.10 a	16.18 ± 1.02 a	4.68 ± 0.38 a	6.26 ± 0.30 a
Average	952 a	1493 a	480 a	242 a	10.08 a	15.20 a	4.37 a	6.10 a
**Late season**								
T_1_	649 ± 15.88 c	941 ± 14.11 d	292 ± 11.82 c	187 ± 10.50 b	5.98 ± 0.58 c	8.51 ± 0.40 c	2.61 ± 0.30 c	4.39 ± 0.22 b
T_2_	994 ± 12.53 a	1430 ± 18.54 b	436 ± 18.32 b	222 ± 7.56 a	9.40 ± 1.02 b	13.46 ± 0.52 b	4.06 ± 0.32 b	5.36 ± 0.30 a
T_3_	970 ± 22.50 b	1364 ± 12.20 c	394 ± 11.50 b	215 ± 12.02 a	9.32 ± .38 b	13.35 ± 1.14 b	4.03 ± 0.82 b	5.37 ± 0.24 a
T_4_	1094 ± 14.00 a	1580 ± 17.32 a	486 ± 21.54 a	240 ± 11.40 a	11.08 ± 1.44 a	15.58 ± 0.92 a	4.50 ± 0.74 a	5.95 ± 0.72 a
T_5_	908 ± 17.77 b	1335 ± 20.52 c	427 ± 11.62 b	209 ± 14.50 a	9.55 ± 1.04 b	14.38 ± 1.10 b	3.88 ± 0.75a	6.05 ± 0.35 a
T_6_	1088 ± 8.25 a	1587 ± 18.02 a	500 ± 10.50 a	205 ± 8.70 a	11.12 ± .42 a	15.60 ± 1.08 a	4.57 ± 0.36 a	5.68 ± 0.30 a
Average	951 a	1459 a	448 a	213 b	9.41 b	13.48 b	4.21 a	5.46 a
**ANOVA**								
Treatment (T)	*	*	*	**	**	**	*	*
Season (S)	ns	ns	ns	*	*	*	ns	ns
T × S	ns	ns	ns	ns	ns	ns	ns	ns

*DMA* dry matter accumulation, *DMT* dry matter translocation, *NA* nitrogen accumulation, *NT* nitrogen translocation, *Ant* anthesis, *Mat* maturity, *Post-a* anthesis accumulation. Values followed by the same letters, within column, are statistically non-significant at (*p* < 0.05). Mean values (n = 3) ± SE. ns: non significant, *p < 0.05, **p < 0.05. For treatment details please see Table 1.

The combination treatments also showed the highest translocation rates of DM and N accumulated pre-anthesis (Table 5). Relative to non-N-treated plots, the combination treatments improved DM and N translocation significantly in both seasons.

### Post-anthesis DM and N accumulation

The combination of manure and synthetic fertilizer significantly improved post-anthesis DM and N accumulation (Table 2). Post-anthesis DM accumulation in the CF-only treatment (T_2_) was 482 and 436 (g m^−2^) in the early and late seasons, respectively, and post-anthesis N accumulation in T_2_ was 4.18 and 4.06 (g m^−2^). Post-anthesis DM accumulation was 9% and 14% higher in the T_6_ combination treatment than in T_2_ in the early and late seasons, respectively, and post-anthesis N accumulation was 10% and 13% higher. T_4_ did not differ significantly from T_6_ in post-anthesis DM and N accumulation. Treatments T_3_ and T_5_ also improved post-anthesis DM and N accumulation, and the lowest post-anthesis values were observed in non-N-treated plots.

### Rice yield and yield components

Rice grain yield and yield attributes were significantly improved by the combination of organic manure and inorganic N fertilization (Table 3). However, there was no significant difference effects of different season on grain yield and yield components, with the exception of panicles number and 1000 grain weight. The T_6_ treatment produced significantly higher panicle number (11% and 14%), grain filling percentage (5% and 7%), 1000-grain weight (6% and 9%), and grain yield (11% and 17%) compared with T_2_ in the early and late seasons, respectively. These parameters did not differ significantly between T_4_ and T_6_. The T_3_ and T_5_ combination treatments also had higher yields and yield components than non-N-treated plots. The lowest yield and yield components were observed in non-N-treated plots.

**Table 3:** Changes in rice growth, yield and yield components under organic and inorganic N fertilizer application.

Treatment	PH (cm)	PN (hill^−1^)	PL (cm)	SSP (panicle^−1^)	FGP (%)	TGW (g)	GY (kg ha^−1^)
**Early season**							
T_1_	90 ± 7.22 c	7.72 ± 1.12 c	20.9 ± 1.55 d	129.23 ± 12.50 d	78.57 ± 4.18 c	18.42 ± 1.85 d	2942 ± 155.52 c
T_2_	105 ± 5.52 b	10.81 ± 1.05 a	25.23 ± 2.40 ab	143.94 ± 9.55 a	84.65 ± 8.10 b	24.52 ± 2.12 a	5038 ± 175.58 a
T_3_	103 ± 8.20 ab	9.60 ± 0.52 b	23.48 ± 2.02 bc	139.33 ± 14.12 b	85.37 ± 7.22 a	23.34 ± 1.82 bc	4276 ± 190.26 b
T_4_	106 ± 10.55 a	10.82 ± 0.32 a	25.47 ± 2.12 a	144.45 ± 10.55 a	85.94 ± 4.52 a	23.58 ± 2.02 ab	5572 ± 265.52 a
T_5_	104 ± 4.58 ab	9.68 ± 1.04 b	23.52 ± 1.86 c	140.73 ± 17.44 b	85.64 ± 3.18 b	22.83 ± 2.30 b	4050 ± 185.50 b
T_6_	105 ± 7.22 a	10.57 ± 1.07 a	25.55 ± 1.10 a	144.05 ± 8.86 a	85.53 ± 3.12 a	24.31 ± 1.12 a	5855 ± 105.40 a
Average	102 a	9.43 b	23.80 a	140.25 a	84.25 a	22.76 b	4576 a
**Late season**							
T_1_	92 ± 4.58 c	6.65 ± 0.90 c	21.02 ± 1.80 d	130.23 ± 10.54 d	77.55 ± 4.62 c	18.31 ± 1.66 d	2855 ± 178.40 d
T_2_	103 ± 12.15 b	9.66 ± 1.04 c	24.65 ± 1.72 ab	143.55 ± 10.50 ab	84.35 ± 4.32 b	23.83 ± 1.10 b	4559 ± 212.58 b
T_3_	103 ± 10.30 b	10.04 ± 0.52 b	23.59 ± 2.08 b	142.03 ± 16.66 bc	85.33 ± 4.40 a	23.52 ± 2.12 b	4197 ± 185.80 c
T_4_	105 ± 8.55 a	10.56 ± 1.10 a	24.86 ± 1.52 a	144.92 ± 15.05 a	85.52 ± 8.10 a	24.64 ± 1.16 a	5251 ± 268.42 a
T_5_	104 ± 6.54 ab	10.16 ± 1.04 b	23.46 ± 1.72 b	142.14 ± 13.30 bc	85.34 ± 4.12 a	23.34 ± 1.14 b	4168 ± 288.52 c
T_6_	106 ± 7.52 a	10.64 ± 1.04 a	25.04 ± 2.04 a	145.44 ± 11.55 a	85.35 ± 5.05 a	24.58 ± 2.20 a	5273 ± 145.50 a
Average	102 a	9.55 a	23.78 a	141.24 a	83.8 a	23.06 a	4383 a
**ANOVA**							
Treatment (T)	*	**	**	**	**	**	**
Season (S)	ns	*	ns	ns	ns	*	ns
T × S	ns	ns	ns	ns	ns	ns	ns

*PH* plant height, *PN* panicle number, *PL* panicle length, *SSP* spikelet number per panicle, *FGP* filled grain percent, *TGW* thousand grain weight, *GY* grain yield. Values followed by the same letters, within column, are not significantly different at *p* ≤ 0.05. Mean values (n = 3) ± SE. ns: non significant, *p < 0.05, **p < 0.05. For treatment details please see Table 1.

### Relationships between leaf physiological traits and grain yield

**Figure 5:**
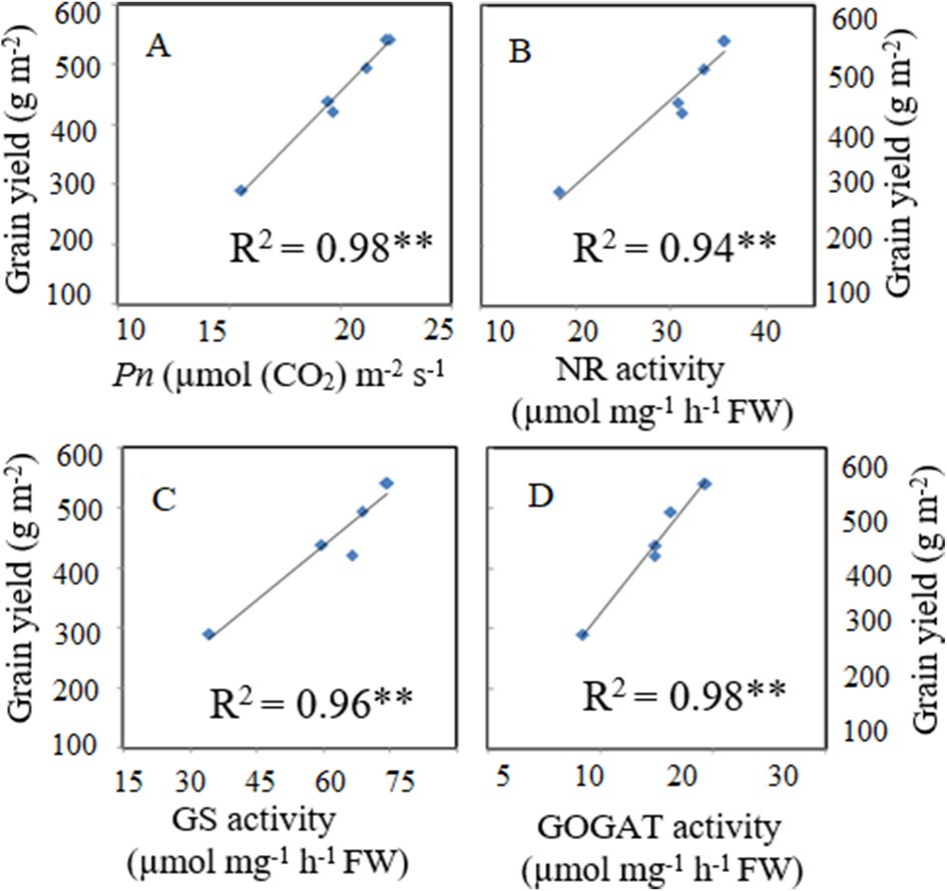
Linear relationships of grain yield with net photosynthetic rate (*Pn*) (**A**), Nitrate reductase (NR) (**B**), glutamine synthetase (GS) (**C**), and glutamine oxoglutarate aminotransferase (GOGAT) (**D**). **p < 0.01. n = 6.

Changes in leaf physiological traits significantly affect the grain yield of rice. In the present study, linear regression analysis revealed highly significant and strong relationships between leaf physiological attributes and grain yield, as shown in Fig. 5. Flag leaf *Pn* (R^2^ = 0.98**, Fig. 5A), and the activities of the N-metabolizing enzymes NR (R^2^ = 0.94**, Fig. 5B), GS (R^2^ = 0.96**, Fig. 5C), and GOGAT (R^2^ = 0.98**, Fig. 5D) were strongly positively associated with grain yield. Therefore, higher leaf physiological activity during the grain filling period contributed significantly to rice grain yield.

### Relationships of pre- and post-anthesis DM and N accumulation with grain yield

**Figure 6:**
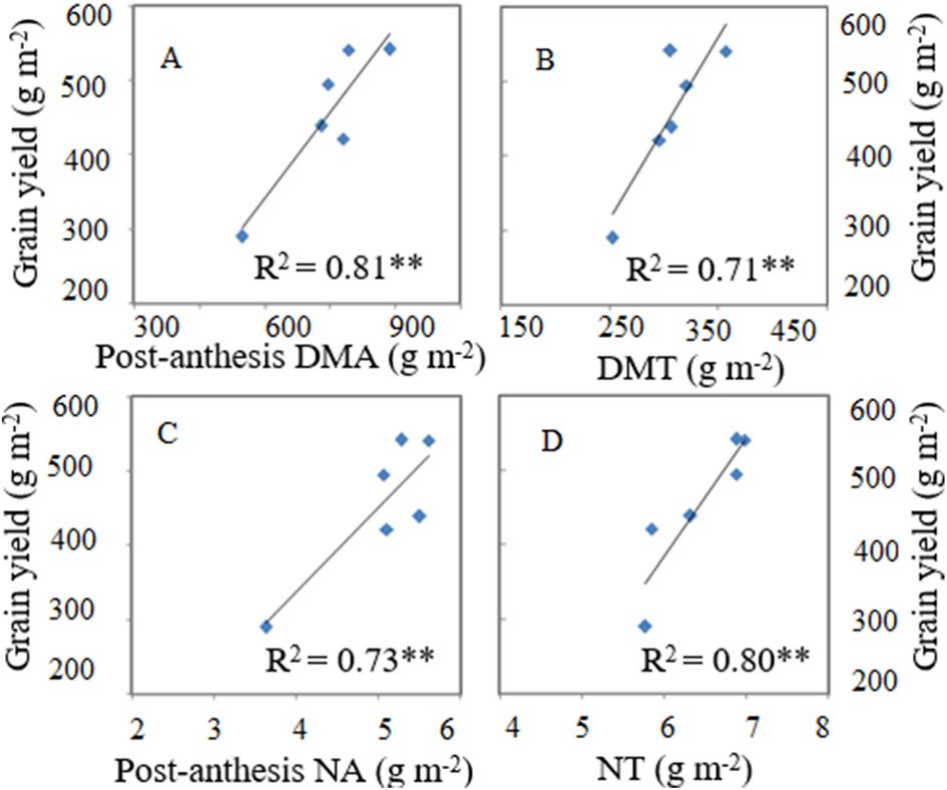
Linear relationships of grain yield with post-anthesis DMA (**A**), dry matter translocation (**B**), post-anthesis NA (**C**), and nitrogen translocation (**D**). Note:DMA- dry matter acuumulation, DMT- dry matter translocation, NA-nitrogen accumulation, NT- nitrogen translocation. **p < 0.001. n = 6.

The grain yield of cereal crops highly dependent on pre- and post-anthesis accumulation of DM and N. In the current study, linear regression analysis showed strong positive relationships of post-anthesis DM (DMA, R^2^ = 0.81**, Fig. 6A) and N accumulation (NA, R^2^ = 0.73**, Fig. 6C) with grain yield. Moreover, translocation of DM (DMT, R^2^ = 0.71**, Fig. 6B) and N (NT, R^2^ = 0.80**, Fig. 6D) accumulated pre-anthesis were also positively related to grain yield. Finally, linear regression confirmed that post-anthesis DM accumulation was strongly positively correlated with grain yield. Therefore, improvements in post-anthesis DM accumulation contributed significantly to higher grain yield in rice.

### Relationships of soil properties with N-metabolizing enzyme activities and grain yield

**Figure 7:**
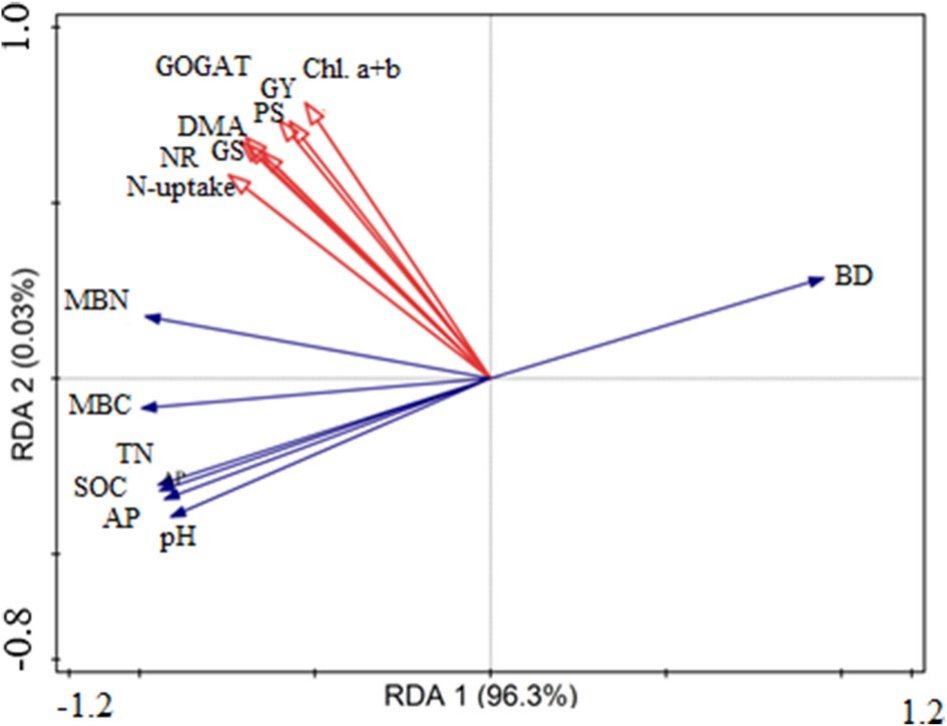
Ordination plot of results from redundancy analysis to identify relationship between soil properties, pH, soil organic carbon (SOC), total nitrogen (TN), available phosphorous (AP) bulk density (BD), and microbial biomass carbon (MBC) and nitrogen (MBN) with N metabolism enzymes activities (NR, GS, and GOGAT), dry matter accumulation (DMA), and grain yield (GY).

Changes in above-average plant yields are highly dependent on fluctuations in soil quality and can be helpful in soil sustainability and stability. In this study, RDA revealed strong positive relationships of N metabolism enzyme activities and grain yield with soil properties and microbial activity (Fig. 7). Soil properties such as pH, SOC, TN, AP, MBC, and MBN showed strong correlations with plant biomass accumulation, rice grain yield and N metabolism enzyme activities, and photosynthetic efficiency during the grain-filling period. However, the correlation of MBN with N metabolism enzyme activity, plant N, and biomass accumulation was significantly higher under organic manure fertilization, presumably as a result of improved soil fertility.

## Discussion

Soil physical and chemical properties were significantly improved by the combination of organic and inorganic N fertilization compared with the application of urea fertilizer alone (Table 1). The combination treatment also resulted in lower soil BD. Reductions in soil BD in the combination treatments were due to the bulky nature of the organic manure, which prevented the soil from separating^34^. Moreover, the use of organic manures has been shown to promote soil aeration and enhance soil aggregation, which leads to a decline in soil BD^35^. Our outcomes are in consistent with Franzluebbers et al.^35^, who concluded that variation in SOC not only directly affects soil BD but also increases soil aggregation and healthy pore growth due to improved soil physicochemical and biological properties.

Soil chemical properties such as soil pH, SOC, TN, AN, AK, and AP increased significantly with the combined application of organic and inorganic fertilizers. We noted that the decomposition of organic manure gradually released nutrients into the soil, and increasing the amount of organic fertilizer from 30 to 60% improved soil chemical properties. The use of CF alone reduced soil pH, whereas the combination treatment increased soil pH considerably. Chemical N fertilization may have significantly reduced the exchangeable base cations in the soil, ultimately leading to a decline in soil pH. The use of synthetic N has also been shown to shift soils into the Al^3+^ buffering stage^36^. Another possible explanation for increased soil pH in the combination treatments is that organic fertilizer contains enough basic cations and carbonate ions to neutralize the acidification effect^37,38^.

Soil C is an important parameter for the evaluation of soil quality and fertility. The substantial improvements in SOC, TN and AN observed in this study may have resulted from both direct C and N inputs from the organic manure and indirect C and N inputs associated with greater crop biomass, such as roots and crop residues^38,39^. Soil organic C at any specific location depends mainly on the seasonal return and recycling of organic materials, roots, and shoot stubbles^40,41^. Our results are in agreement with Purakayastha et al.^42^, who reported that organic manure integrated with chemical fertilizer increased soil TN by 56–90% and SOC by 11–80% in the upper soil layer. The application of organic fertilizers may also have increased soil nutrient availability because the manure absorbed more leachate, improving soil water holding capacity, decreasing nutrient leaching, and ultimately increasing the availability of N, P, and K in the soil^43^.

Higher soil available P in the combination treatments is consistent with the results of P addition from chemical fertilizers, as plants typically use only a portion of the applied P^44^. Likewise, organic manure often provides a large amount of P to the soil and reduces the fixation of added P, resulting in enhanced competition of organic molecules with PO_4_^3−^ ions for P retention sites in the manure treatments^45^. Higher available K in the combination treatments relative to the urea-only treatment may reflect exudation of organic acids during the decomposition process, which releases negative ions that have a preference for di- or trivalent cations (e.g., Al^3+^, Ca^2+^, and Mg^2+^), leaving K^+^ to be absorbed by negatively charged soil colloids^46^. This phenomenon can help to minimize K fixation and improve soil K availability.

Soil MBC and MBN reflect characteristics of the soil microbial community structure^47^. In the present study, organic manure in combination with CF significantly enhanced soil MBC and MBN (Fig. 1). Increases in MBC and MBN may have occurred because organic fertilizer improved the physicochemical and biological properties of the soil, leading to increased absorption and uptake of mineral N by the crop^48,49^. In addition, manure may have facilitated the conversion of mineral N to MBN and other N forms^50^. Another possible explanation is that the combination treatments may have improved soil fertility and rice biomass production (Tables 4, 5), leading to an increase in crop residues. Such residues are beneficial for the propagation of soil microbes and may therefore facilitate the conversion of C and N^51^. Furthermore, the integration of organic fertilizer with synthetic N is widely accepted as an efficient means of improving soil microbial activity, soil structure, aggregation, and water retention capacity^13,14^.

**Table 4:** Mean maximum and minimum temperatures, relative humidity, and total rainfall during both growing seasons.

Months	Maximum Temperature (°C)	Minimum Temperature (°C)	Relative Humidity (%)	Total Rainfall (mm)
March	24	16	83	72
April	29	21	76	92
May	31	23	83	176
June	35	25	81	211
July	35	26	82	231
August	36	26	80	151
September	33	24	79	115
October	29	21	82	98
November	26	16	87	110

**Table 5:** Physical and chemical properties of soil and manure before the experimentation.

Properties	Soil	Cattle Manure	Poultry Manure
Porosity (%)	40.52	–	–
Moisture content (%)	11.93	–	–
Bulk density (g cm^−3^)	1.36	0.81	0.74
pH (water)	5.94	7.75	7.95
SOC (g kg^−1^)	14.56	146.33	164.22
SOM (g kg^−1^)	25.08	254.63	282.42
Total N (g kg^−1^)	1.41	9.76	13.58
Total P (g kg^−1^)	0.75	10.12	7.32
Total K (g kg^−1^)	-	14.22	9.76
Available N (mg kg^−1^)	134.7	–	–
Available P (mg kg^−1^)	23.12	–	–
Available K (mg kg^−1^)	233.3	–	–

*SOC* soil organic carbon, *SOM* soil organic matter, *N* nitrogen, *P* phosphorous, *K* potassium, *C: N* carbon to nitrogen ratio.

Leaf chlorophyll (Chl) content is widely used to assess plant photosynthetic health^52^. Chl synthesis and protein are also associated with leaf N concentration, and higher photosynthetic rates promote stem elongation, enhance leaf area expansion, and delay leaf senescence^53^. Leaf Chl content and *Pn* are directly related to N uptake^54^. Photosynthetic rate responds readily to N and water supply and is the key driver of plant production by enhancing growth and biomass^55,56^. In this work, leaf *Pn* and Chl content were highest in the integration treatments relative to the CF-only application during the grain-filling (Figs. 3 and 4). This increase may have been due to the fact that the combined application of manure and synthetic fertilizer increased soil fertility and quality (Table 4), decreasing the leaching of mineral elements from the upper soil layer and improving the physical structure of the soil, thereby increasingplant nutrient absorption^57^. Another possible reason for improvement in *Pn* and Chl content during the grain-filling period is the quicker release of nutrients from synthetic fertilizer and accompanied by the slow and steady release of nutrients from organic fertilizer across the entire growth period^20^. *Pn* and Chl a were always highest in T_6_ and lowest in T_1_ (Figs. 3 and 4). These findings highlight the importance of Chl a, as it is the primary photosynthetic pigment.

Several key enzymes such as NR, GS, and GOGAT play an important role in plant N assimilation^27^. In this study, higher N-metabolizing enzyme activity was observed in response to the combined application of manure and synthetic fertilizer (Fig. 4). This may have resulted from improved soil quality under the combination treatment (Table 5). Our findings indicated that the combination of organic manure and chemical fertilizer improved N accumulation in leaves more effectively than the traditional urea-only application, and this was necessary to provide adequate grain-filling substrate and promote superior grain yield^58^. Similarly, Gupta et al.^59^ also reporteda strong relationship of soil N availibility and N absorption with the activities of key N assimilatory enzymes during grain filling. Our outcomes are consistent with those of Sun et al.^58^, who concluded that GS and GOGAT activities in flag leaves during the grain-filling period were strongly positively associated with grain yield and crop quality.

Delayed leaf senescence supports comparatively high photosynthetic activity and promotes maximum DM production and grain yield; it may be achieved by synchronizing soil N availability and plant N uptake during the grain-filling period^24,25^. In the present study, delayed leaf senescence, high photosynthetic efficacy, and enhanced N-metabolizing enzyme activity were observed during the grain-filling period in the combination treatments (Figs. 3 and 4). These treaits improved N uptake and post-anthesis DM accumulation, and ultimately improved grain yield. This was further supported by the significant and highly positive correlations of grain yield with Pn (R^2^ = 0.98**, Fig. 5A), NR (*R*^*2*^ = 0.94**, Fig. 5B),GS (*R*^*2*^ = 0.96**, Fig. 5C) and GOGAT (*R*^*2*^ = 0.98**, Fig. 5D), indicating that greater *Pn* and N metabolism activities during grain formation promoted the formation of a superior sink (as quantified by panicle length and spikelet number), and consequently led to superior rice grain yield. Consistent with our results, many authors have reported that increased N uptake leads to increases in *Pn*, overall photochemical efficiency of PSII, and leaf physiological activity; this delays leaf senescence in the late growth period and eventually enhances photosynthetic production during the grain-filling stage^59,60^.

Cereal crop yields are strongly dependent on post-anthesis DM production and the translocation of DM accumulated prior to anthesis to grain^30^. Pal et al.^30^ also concluded that the contribution to grain yield of DM production prior to anthesis ranged from 22 to 69%, depending on rice cultivar and the sowing time. Moreover, Wu et al.^32^ stated that variation in rice yield between the early and late growing seasons could be explained primarily by the difference in post-anthesis DM production. In the current study, the combined treatments T_4_ and T_6_ had the highest values of DMT and accumulated pre-anthesis and post-anthesis DM production, as shown in Table 2. Highest DM and N accumulation under the combination of 30% manure and 70% inorganic fertilizer could be attributed to a high and continuous supply of nutrients due to improved soil fertility (Table 1). The constant and steady release of N from the cattle and poultry manure, particularly during the grain-filling period, would have encouraged their use by the plant^26^.

Linear regression analysis showed that post-anthesis DM accumulation was more strongly positively correlated with grain yield (*R*^*2*^ = 0.81**, Fig. 6A) than was DMT (*R*^*2*^ = 0.71*, Fig. 6B). This finding underscored that both processes are important, but post-anthesis DM production plays a more important role in higher grain yield. Similarly, pre- and post-anthesis N accumulation were also highly positively correlated with grain yield: post-anthesis NA (*R*^*2*^ = 0.73**, Fig. 6C) and NT (*R*^*2*^ = 0.80**, Fig. 6D). The current study confirms that plants rely primarily on post-anthesis DM production and N accumulation for grain filling. Higher post-anthesis DM production and N accumulation in the combination treatments were due mainly to adequate availability of nutrients, which delayed leaf senescence and increased N remobilization.

Rice grain yield is determined by yield components, including the number of tillers, panicle length, and spikelets per panicle^32,61^. In the present study, the combination of manure and synthetic N fertilizer significantly improved rice growth, yield, and yield components compared to urea fertilization alone (Table 3). The higher rice growth, yield, and yield traits under the combination treatments could be attributed mainly to a balanced and continuous supply of nutrients due to enhanced soil fertility (Table 4), which ultimately improved plant nutrient uptake and growth. The continued and slow release of N from organic manure, particularly during the grain-filling period, may have enabled its efficient utilization by the crop^41,62^. Moreover, the RDA showed that the x-axis explained 96.3% of the variation, and the y-axis explained 0.03%. It revealed significant positive correlations of grain yield, leaf physiological traits, N metabolism enzyme activities, and dry matter accumulation with all soil properties (Fig. 7).

## Conclusion

Application of a combination of organic manure and chemical fertilizer enhanced soil physical and biochemical traits, leaf physiological activity, and rice yields compared with chemical fertilizer alone. The co-incorporation of manure and synthetic fertilizers significantly improved pre- and post-anthesis DM production and N accumulation compared with the application of urea alone. Improvements in DM production and N accumulation were due primarily to improved soil fertility and leaf physiological activity, including *Pn*, Chl, and the activities of N-metabolizing enzymes, which further increased DM production and N uptake. RDA revealed positive relationships between grain yield and soil properties (i.e., SOC, TN, AN, and AP), and a significantly higher correlation was observed between grain yield and soil MBC and MBN. The combination of organic manure and synthetic fertilizer in a 30:70 ratio is a beneficial and sustainable practice for rice production and soil quality improvement.

## Materials and methods

### Experimental site

The experiment was conducted at the experimental farm of Guangxi University, China (22° 49′ 12″ N, 108° 19′ 11″ E; 75 m) in the early season (March to July) and late season (July to November) of 2019. The region is characterized by a subtropical, monsoon climate with average annual rainfall of 1080 mm. The average maximum and minimum temperatures are 32.5 °C and 24.2 °C in the early season and 31.2 °C and 22.0 °C in the late season (Table 4). The soil is classified as an ultisol based on USDA classification. It is slightly acidic with pH 5.94, soil organic carbon (SOC) 14.56 g kg^−1^, total N (TN) 1.41 g kg^−1^, available phosphorous (P) 23.12 mg kg^−1^, available potassium (K) 233.33 mg kg^−1^, and a high bulk density (BD) of 1.36 g cm^−3^ (Table 5).

### Experimental design and field management

The field experiment was performed in a randomized complete block design (RCBD) with three replicates and a plot size of 3.9 m × 6 m (23.4 m^2^). Cattle manure (CM) and poultry manure (PM) were the organic fertilizers and urea was the chemical fertilizer (CF). The experiment consisted of six treatments, i.e., : no N fertilizer (T_1_); 100% CF (T_2_); 60% CM + 40% CF (T_3_); 30% CM + 70% CF (T_4_); 60% PM + 40% CF (T_5_), and 30% PM + 70% CF (T_6_). The noodle rice cultivar “Zhenguiai” was used as the test crop. Rice seeds were sown in an open filed in plastic seedling trays, and urea was applied to the nursery at the time of preparation. The 25 day- old seedlings were transplanted to the field, and two seedlings per hill . The plant-to-plant distance was 10 cm, the row-to-row distance was 30 cm, and the total number of plants in each plot was 780. The recommended dose of NPK was 150:75:150 (kg ha^−1^), and every plot received 175.5 g of P_2_O_5_ from superphosphate, 365 g of KCl from potassium chloride, and 351 g of N from PM or CM and CF (urea) after proper N estimation. The net N, P, and K contents in the urea, superphosphate, and potassium chloride was 46%, 20%, and 60%, respectively. The chemical composition of the organic manure and the nutrient content and quantity for each treatment are shown in Tables 5 and 6. N and KCI were applied in three splits as a basal dose (60%), at the early tillering stage (20%), and at panicle initiation (20%). P was delivered as a basal dose one day before transplantation (Table 6).

**Table 6:** Nutrient content and amount nutrient provided of each treatment and application time.

Treatment	N (g plot^−1^)	Urea (g plot^−1^)	CM or PM (kg plot^−1^)	Basal fertilization (kg plot^−1^)	Tillering (g plot^−1^)	Panicle initiation (g plot^−1^)
T_1_: No− N	0	0	0	P_2_O_2_: 0.93, KCl: 0.30	KCl: 0.30	Urea: 0
T_2_: 100% CF	351	753	0	Urea: 0.45, P_2_O_2_: 0.93, KCl: 0.30	Urea: 150, KCl: 0.30	Urea: 150
T_3_: 60% CM + 40% CF	351	301	21.5	Urea: 0, CM: 21.5, P_2_O_2_: 0.93, KCl: 0.30	Urea: 150, KCl: 0.30	Urea: 150
T_4_: 30% CM + 70% CF	351	527	10.7	Urea:1.17, CM: 10.7, P_2_O_2_: 0.93, KCl: 0.30	Urea: 150, KCl: 0.30	Urea: 150
T_5_: 60% PM + 40% CF	351	301	15.5	Urea: 0, PM: 15.5, P_2_O_2_: 0.93, KCl: 0.30	Urea: 150, KCl: 0.30	Urea: 150
T_6_: 30% PM + 70% CF	351	527	7.7	Urea: 0, PM: 7.7, P_2_O_2_: 0.93, KCl: 0.30	Urea: 150, KCl: 0.30	Urea: 150

*N* nitrogen, *CF* chemical fertilizer (urea), *CM* cattle manure, *PM* poultry manure, *P*_*2*_*O*_*2*_ superphosphate, *KCl* potassium chloride.

Organic fertilizer, such as CM and PM were obtained from the cattle and poultry farms, located in the local area. Organic manure applied to plots 20 days prior to transplantation. The T_1_ treatment received no N fertilizer but received P and K fertilizers at rates equal to those in N-treated plots. Standard flood water was provided at a depth of approximately 5 cm from transplant to physiological maturity. Normal agricultural practices were used for all treatments, including irrigation (about 5 cm flood water), insecticide application (chlorantraniliprole formulations sprayed at the recommended rate of 150 mL a.i. per ha), and herbicide application (paraquat at 10 gallons per acre).

### Sampling and analysis

#### Sampling and analysis of soil and manure

Subsamples of initial soil and organic fertilizers (CM and PM) were dried at room temperature and passed through a 2-mm sieve. Inaddition, three replicate soil samples were taken from the 0–20 cm depth after harvest in the early and late seasons, to assess changes in soil properties. Soil bulk density (BD) was determined by the core method as described by Grossman^63^, and used to calculate soil total porosity using Eq. (1) recomended by Hillel^64^:

1 $${\text{Porosity }} = \, \left( {1 - \left( {{\text{BD}}/{\text{PS}}} \right)} \right) \, \times \, 100$$

where BD is soil bulk density and PS is particle density, assumed to be 2.65 mg m^−3^. Soil moisture content was measured as described by Ledieu et al^65^. Air-dried soil was passed through a 0.5-mm sieve, and the weight of the tin (g) was taken as W1. A 1 g soil sample was taken along with the tin and weighed as W2. The soil samples were dried in an oven for 2 h at 105 °C to obtain a constant weight as W3. Soil moisture content (%) was determined using Eq. (2):

2 $${\text{MC }}\% = \frac{{{\text{W}}2 - {\text{W}}3}}{{{\text{W}}3 - {\text{W}}1 }}$$

Soil organic carbon was measured using the oxidation method. Soil subsamples (0.5 g) were digested with 5 mL of 1 M K_2_Cr_2_O_7_− H_2_SO_4_ and 5 mL of concentrated H_2_SO_4_ and boiled at 175 °C for 5 min, accompanied by titration of FeSO_4_ digests according to the method of Bao^66^. To measure total soil N content, 200 mg of soil was digested using the salicylic-acid sulfuric-acid hydrogen peroxide method of Ohyama et al.^67^, and TN was analyzed using the micro-Kjeldahl method of Jackson^68^. Total P was determined using the ascorbic acid described by Murphy^69^. Total K was measured by preparing a standard stock solution by dissolving KCI in distilled water and measuring TK at 7665 R wavelength with an atomic absorption spectrophotometer (Z-5300; Hitachi, Tokyo, Japan) after sample digestion. Soil organic matter (SOM) was calculated by multiplying the SOC by 1.72.

AN was estimated using the methods of Kostechkas and Marcinkevicinee^70^ and Dorich and Nelson^71^. Soil AP was measured by the NaHCO_3_ extraction method and analyzed by the Mo-Sb colorimetric procedure using a spectrophotometer (UV 2550, Shimadzu, Japan) by method of Bao^66^. Soil AK was assessed by the method of Knudsen et al.^72^, using normal 1 M NH_4_OAc. Soil pH was measured with a digital pH meter (Thunderbolt PHS-3C, Shanghai, China) after mixing the soil and organic manure with distilled water at a 1:2.5 (w/v) ratio for 1 h.

### Soil microbial biomass

The fumigation extraction technique was used to determine MBC as described by Brookes et al.^73^, and MBN according to the procedure of Vaince et al.^74^. From the composite soil samples, we took 10 g of soil and divided it into similar halves. In a vacuum desiccator, 25 ml of ethanol-free CHCL_3_ was put in petri dish to disinfect first half of the soil (5 g) for 24 h at room temperature (25 °C). The samples were placed in warm water at 80 °C after fumigation to eliminate fumes, and 20 ml of K_2_SO_4_ (0.5 M) was then used to remove C and N from both the fumigated and non-fumigated soils. The filtrated samples were then processed on a TOC Analyzer (TNM1; Shimadzu) and subjected to Kjeldhal digestion in order to calculate total C (TC) and TN. Equation (3) was used to estimate MBC and MBN:

3 $$MBC \;or\; MBN = \frac{TN\; or\; TCfu\; - \;TN or\;TCnfu}{{kEN\; or\;kEC}}$$

where TNfu and TNnfu are the total N in fumigated and non-fumigated samples, and TCfu and TCnfu are the TC concentrations in fumigated and non-fumigated samples. A kEC coefficient of 0.45 was used to estimate MBC according to the method of Jenkinson et al.^75^, and a kEN coefficient of 0.54 was used to estimate MBN according to the method of Joergensen and Mueller^76^.

### Accumulation and translocation of DM and N

Three replicate plants were collected at anthesis and at physiological maturity to measure DM and N accumulation. The rice plant was divided into leaves (leaf blade + leaf sheeth), stems, and panicles, and then oven-dried to constant weight at 65 °C. Rice plant sub samples were ground to powder, and total N was estimated using the micro-Kjeldhal method as described by Jackson^68^. Post-anthesis DM or N accumulation is considered to be the difference in aboveground accumulation between anthesis and maturity^30^. Assuming that all DM and N losses from the vegetative organs of the plant were transferred to the grains, N translocation (NT) and DM translocation (DMT) during the grain filling stage were measured according to the equations of Papakosta and Gagianas^77^:

4 $$DMT = DMa - \left( {{\text{DMleaf}},{\text{m}} + {\text{DMstem}},m + DMchaff,m} \right)$$

5 $$NT = NTa - \left( {{\text{NTleaf}},{\text{m}} + {\text{NTstem}},m + NTchaff,m} \right)$$

where DMa is the total aboveground DM accumulation at anthesis and DM_stem,m_, DM_leaf,m_, and DM_chaff,m_ are the DM of leaves, stems, and chaff at maturity. NTa is the total aboveground N accumulation at anthesis, and NT_stem,m_, NT_leaf,m_, and NT_chaff,m_ are the total N accumulation of stems, leaves, and chaff at physiological maturity.

### Rice leaf net photosynthetic efficiency and chlorophyll content

To assess the process of leaf senescence during the reproductive phase, flag leaf chlorophyll content, photosynthetic rate (*Pn*), and days to maturity were also determined. Flag leaf *Pn* and Chl content (Chl a and Chl b) were measured at the grain-filling stage. *Pn* was measured on the completely expanded flag leaf using a portable photosynthesis instrument (LI-6400, LI-COR, Lincoln, NE, USA). Measurements were made on a sunny day from 10:00 a.m. to 12:00 p.m. The sampling conditions were light intensity 1200 µmol m^−2^ s^−1^, air humidity 70%, CO_2_ 375 μmol mol^−1^, and leaf temperature 28 °C.

To measure leaf chlorophyll content, 1 g of fresh leaf tissue was cut into small pieces, placed in a volumetric flask that contained 10 mL of 80% acetone solution as described in Porra et al.^78^, and stored in the dark for 24 h. The absorbance of the extracted solution was measured at 663 and 645 nm using a UV spectrophotometer (Infinite M200, Tecan, Männedorf, Switzerland) to estimate chlorophyll a and b content (mg g^−1^) using the equations described by Arnon^79^:

6 $${\text{C}}_{{({\text{Chl a}})}} = \, 12.71\;{\text{D}}_{663} - 2.59{\text{ D}}_{645}$$

7 $${\text{C}}_{{({\text{Chl b}})}} = \, 22.88{\text{ D}}_{645} - 4.67{\text{ D}}_{663}$$

where C_(Chl a)_ and C_(Chl b)_ are the content of Chla or Chb; D_663_ and D_645_ are the absorbance at 663 and 645 nm, respectively, using spectrophotometer (Model-1800, Tecan-infinite M200, Switzerland).

### Nitrogen metabolizing enzyme activities 

Five flag leaves were collected from each treatment during the grain-filling period, immediately frozen in liquid N and stored at ‒ 80 °C for estimation of the activities of the N-metabolizing enzymes such as Nitrate reductase (NR), Glutamine synthetase (GS), and Glutamate synthase was extracted and measured using a Glutamate Synthase (GOGAT).

NR was extracted and measured using a Nitrate Reductase (NR) assay Kit (BC0080, Solarbio, Beijing, China). Briefly, 0.1 g leaf samples were extracted in 1 ml extraction solution and the mixture was centrifuged at 4000 g for 10 min. The resulting supernatant was collected for further analysis. The absorbance at 520 nm was used for the calculation of NR activity. GS was extracted and measured using a Micro Glutamine Synthetase (GS) assay Kit (BC0915, Solarbio, Beijing, China). Briefly, 0.1 g leaf samples were thoroughly ground in liquid nitrogen and extracted with 1 mL extraction buffer. The mixture was centrifuged at 8000 g at 4 °C for 10 min. The supernatant after centrifuging was collected for activity measurement. The absorbance at 520 nm was used for the calculation of GS activity.

Glutamate synthase was extracted and measured using a Glutamate Synthase assay Kit (BC0070, Solarbio, Beijing, China). Briefly, 0.1 g leaf samples were extracted in 1 mL extraction buffer. The extraction mixture was centrifuged at 10,000 g at 4 °C for 10 min. The resulting supernatant was harvested and the absorbance at 340 nm was measured for the calculation of GOGAT activity.

### Rice yield and yield attributes

Five central rows from each plot were selected at physiological maturity to measure rice growth, yield, and yield traits. Three hills were randomly selected at physiological maturity to measure plant height, panicle number, panicle length, spikelet number per panicle, grain filling percentage, and thousand-grain weight. The crop was harvested when almost all the heads showed a complete loss of green color. Grain yield (kg/ha) was measured from five central rows in each treatment and adjusted to 14% moisture content.

### Statistical analysis

Analysis of variance (ANOVA) was performed with Statistics 8.1 software to examine variations in soil physical and biochemical properties, leaf physiological traits, rice grain yield, and yield components. Percentage data were arcsine transformed prior to analysis. Data from both seasons were used in the analysis in order to detect differences between seasons as well as fertilizer treatments. Treatment was considered to be a fixed effect, and season was considered to be a repeated measures factor and a fixed effect. The interaction between fertilizer treatment and season was also taken as a fixed effect, but the interaction of season and treatment with replication was taken as a random effect. Mean separation was performed using the least significant difference (LSD) method at* p* < 0.05. Linear regression analysis was performed to evaluate the relationship between grain yield and *Pn*, N-metabolizing enzyme activities, pre-and post-anthesis DM, and N accumulation. Redundancy analysis (RDA) was performed using Canoco version 5 (Cambridge University Press, Cambridge, UK). .

## Abbreviations

N: Nitrogen
CF: Chemical fertilizer
OM: Organic manure
CM: Cattle manure
PM: Poultry manure
NR: Nitrate reducates
GS: Glutamine synthetase
GOGAT: Glutamate oxoglutarate aminotransferase
SOC: Soil organic carbon
TN: Total nitrogen
BD: Bulk density
DM: Dry matter
DMT: Dry matter translocation
NT: Nitrogen translocation
*Pn*: Photosynthetic rate
DAA: Days after anthesis
Chl.a: Chlorophyll a
Chl.b: Chlorophyll b
MBC: Microbial biomass carbon
MBN: Microbial biomass nitrogen
