# Author Correction: Co-incorporation of manure and inorganic fertilizer improves leaf physiological traits, rice production and soil functionality in a paddy field

**DOI:** 10.1038/s41598-021-96497-z

**Published:** 2021-08-17

**Authors:** Anas Iqbal, Liang He, Izhar Ali, Saif Ullah, Aziz Khan, Kashif Akhtar, Shangqin Wei, Shah Fahad, Rayyan Khan, Ligeng Jiang

**Affiliations:** 1 College of Life Science and Technology, Guangxi University, Nanning, 530004 China; 2 Key Laboratory of Crop Cultivation and Farming Systems College of Agriculture, Guangxi University, Nanning, 530004 China; 3 Institute of Nuclear Agricultural Sciences, College of Agriculture and Biotechnology, Zhejiang University, Hangzhou, 310058 China; 4 Department of Agronomy, The University of Haripur, Khyber Pakhtunkhwa, Pakistan; 5 Tobacco Research Institute, Chinese Academy of Agricultural Science, Qingdao, 266101 China

Correction to: *Scientific Reports* 10.1038/s41598-021-89246-9, published online 11 May 2021

The original version of this Article omitted an affiliation for Anas Iqbal and Ligeng Jiang.

The correct affiliations for Anas Iqbal are listed below:

College of Life Science and Technology, Guangxi University, Nanning 530004 China

Key Laboratory of Crop Cultivation and Farming Systems College of Agriculture, Guangxi University Nanning 530004, China

Department of Agronomy, The University of Haripur, Khyber Pakhtunkhwa, Pakistan

The correct affiliations for Ligeng Jiang are listed below:

College of Life Science and Technology, Guangxi University, Nanning 530004 China

Key Laboratory of Crop Cultivation and Farming Systems College of Agriculture, Guangxi University Nanning 530004, China

As a result, Affiliations 2-5 were incorrectly listed as Affiliation 1-4.

The original Article has been corrected.

